# Validity of estimating minute-by-minute energy expenditure of continuous walking bouts by accelerometry

**DOI:** 10.1186/1479-5868-8-92

**Published:** 2011-08-24

**Authors:** Erin E Kuffel, Scott E Crouter, Jere D Haas, Edward A Frongillo, David R Bassett

**Affiliations:** 1 Department of Health, Exercise & Rehabilitative Services, PO Box 5838, 175 W Mark Street, Room 358 Maxwell, Winona, MN 55987 USA; 2 Dept. of Exercise and Health Sciences, University of Massachusetts, 100 Morrissey Blvd, Boston, MA 02125 USA; 3 Division of Nutritional Sciences, 211 Savage Hall, Cornell University, Ithaca, NY 14853 USA; 4 Department of Health Promotion, Education, and Behavior, Arnold School of Public Health, 800 Sumter Street, Room 216, University of South Carolina, Columbia, South Carolina 29208 USA; 5 Department of Exercise, Sport, and Leisure Studies, The University of Tennessee, 1914 Andy Holt Avenue, Knoxville, TN 37996-2700 USA

## Abstract

**Background:**

Objective measurement of physical activity remains an important challenge. For wearable monitors such as accelerometer-based physical activity monitors, more accurate methods are needed to convert activity counts into energy expenditure (EE).

**Purpose:**

The purpose of this study was to examine the accuracy of the refined Crouter 2-Regression Model (C2RM) for estimating EE during the transition from rest to walking and walking to rest. A secondary purpose was to determine the extent of overestimation in minute-by-minute EE between the refined C2RM and the 2006 C2RM.

**Methods:**

Thirty volunteers (age, 28 ± 7.7 yrs) performed 15 minutes of seated rest, 8 minutes of over-ground walking, and 8 minutes of seated rest. An ActiGraph GT1M accelerometer and Cosmed K4b^2 ^portable metabolic system were worn during all activities. Participants were randomly assigned to start the walking bout at 0, 20, or 40 s into the minute (according to the ActiGraph clock). Acceleration data were analyzed by two methods: 2006 Crouter model and a new refined model.

**Results:**

The 2006 Crouter 2-Regression model over-predicted measured kcal kg^-1 ^hr^-1 ^during the first and last transitional minutes of the 20-s and 40-s walking conditions (P < 0.001). It also over-predicted the average EE for a walking bout (4.0 ± 0.5 kcal kg^-1 ^hr^-1^), compared to both the measured kcal kg^-1 ^hr^-1 ^(3.6 ± 0.7 kcal kg^-1 ^hr^-1^) and the refined Crouter model (3.5 ± 0.5 kcal kg^-1 ^hr^-1^) (P < 0.05).

**Conclusion:**

The 2006 Crouter 2-regression model over-predicts EE at the beginning and end of walking bouts, due to high variability in accelerometer counts during the transitional minutes. The new refined model eliminates this problem and results in a more accurate prediction of EE during walking.

## Background

Accelerometers, such as the ActiGraph or Actical, are often used to provide an objective record of physical activity. However, conventional methods of analyzing accelerometer count data have used a single linear regression equation to convert counts into energy expenditure (EE) [1-3]. These single regression equations are developed specifically for each accelerometer brand due to differences in how companies convert the raw acceleration into counts. In general, single linear regression equations, regardless of which brand they were developed for, that are developed on walking and running are valid for those activities, but they underestimate EE for most other types of moderate and vigorous physical activity and overestimate light physical activity [4,5]. Due to the inherent errors in single regression models, researchers have sought to develop other approaches for analyzing accelerometer data.

One such approach, developed for use with the ActiGraph accelerometer, is the 2006 Crouter 2-regression model (C2RM), which does not assume a linear relationship between mean counts and metabolic equivalents (METs) for all activities [6]. Instead, it examines the variability among six consecutive 10-s epochs within a one-minute period to predict EE. If there is a low variability among the 10-s epochs, a walk/run regression equation is used, but if the variability among 10-s epochs is high, a different equation is used for intermittent lifestyle activities.

The 2006 C2RM, for the ActiGraph accelerometer, was an improvement over previous ActiGraph single regression equations [1-3] because it predicted EE more accurately across a wide range of activities [6]. However, a potential problem is that the 2006 C2RM examines six consecutive 10-s epochs and therefore it assumes that an individual is performing an activity for the entire minute on the ActiGraph clock. However, if a walking bout were to start or stop in the middle of a minute, that minute would exhibit high variability in accelerometer counts since rest and walking would be included within the same minute. Thus, the transitional minute could be misclassified as an intermittent lifestyle activity, resulting in an over-prediction of EE due to the use of the wrong regression equation.

Therefore, a refinement of the 2006 C2RM, for the ActiGraph accelerometer, has been proposed [7]. The refined C2RM [7] is based on similar principles to the 2006 C2RM [6], but the refined method allows for any continuous walking bout lasting 60 seconds or longer to be accurately determined, regardless of where it starts or stops on the ActiGraph clock. Thus, a major difference between the refined C2RM and the 2006 C2RM is that EE is estimated every 10 seconds, rather than every 60 seconds allowing for a more accurate prediction of EE. However, the refined C2RM has not been rigorously tested under various walking conditions.

The purpose of this study was to examine the accuracy of the refined C2RM [7] for estimating EE during the transition from rest to walking, and back to rest. A secondary purpose was to determine the extent of overestimation in minute-by-minute EE between the refined C2RM [7] and the 2006 C2RM [6]. We hypothesized that the refined C2RM would eliminate the overestimation of EE that occurs with the 2006 model, during transitions from rest to walking, and back to rest. We also hypothesized that the refined C2RM would predict minute-by-minute EE more accurately than the 2006 C2RM.

## Methods

### Participants

Thirty participants from the University of Tennessee, Knoxville and surrounding community volunteered to take part in this study. The study was approved by the University of Tennessee Institutional Review Board and the Cornell University Committee on Human Subjects. All participants were informed of potential risks and benefits of the study before they provided written informed consent. A health history questionnaire was completed and participants were excluded if they were pregnant or had any contraindications to exercise. Height and body mass (in light clothing and no shoes) were measured using a stadiometer and physician's scale, respectively. Body mass index (BMI) was calculated according to the formula: body mass in kilograms (kg) divided by height squared in meters (m^2^).

### Protocol

Each participant was randomly assigned to one of three pre-planned conditions: activities starting at 0, 20, or 40 s after the minute on the ActiGraph clock. Based on the condition assignment, the participant started the activity at the appropriate time corresponding to the ActiGraph GT1M clock, which was synchronized with a digital stopwatch. For example, if the participant was assigned to the 0-s condition, the participant started and stopped each activity exactly on the minute of the ActiGraph clock, the 20-s and 40-s conditions started and stopped each activity exactly 20 s and 40 s after the minute, respectively. For each condition, the participant performed 15 minutes of seated rest, 8 minutes of continuous walking at a self-selected pace around the perimeter of a gymnasium, and 8 minutes of seated rest. A 5-s countdown was given so the participant started the walking bout at the correct time on the ActiGraph clock. The distance was measured with a measuring wheel for an accurate measurement of total distance walked.

Each participant wore an ActiGraph GT1M accelerometer on the right hip, and oxygen consumption (VO_2_) was measured with a Cosmed K4b^2^, for all activities. All trials took place at the University of Tennessee, Knoxville.

### ActiGraph GT1M

The ActiGraph GT1M (ActiGraph, Pensacola, Florida) is a uniaxial accelerometer. It was initialized using 1-s epochs and synchronized with a digital clock, so the start time could be synchronized with the Cosmed K4b^2^. During the testing it was attached to a nylon belt and positioned on the right hip at the anterior axillary line. Three GT1M accelerometers were used and were calibrated by the manufacturer prior to the start of the study. At the completion of the testing, the data were downloaded to a laptop computer and then converted to 10-s epochs using a Visual Basic program.

### Cosmed K4b^2^

The Cosmed K4b^2 ^(Cosmed, Rome, Italy) is a portable indirect calorimeter that weighs 1.6 kg (including the battery pack and harness). Each participant wore the Cosmed K4b^2 ^to collect breath-by-breath respiratory data. During testing, the Cosmed K4b^2 ^was attached to the participant's chest with the use of the manufacturer's harness. Each participant was fitted with a rubber facemask and disposable gel seal (Hans-Rudolph, Kansas City, MO) to prevent air leaks. The Cosmed K4b^2 ^was calibrated according to the manufacturer's guidelines before each trial. All data were stored in the Cosmed K4b^2 ^during the trials and then downloaded to a laptop computer.

### Data Management

Data for one participant were excluded due to problems encountered when downloading the data. Therefore, one other participant performed two separate conditions (0-s and 40-s conditions) to ensure that we had 10 participants per condition for analyses.

Breath-by-breath data from the Cosmed K4b^2 ^were averaged over 1-minute. Body mass was adjusted for the weight of the ActiGraph GT1M and Cosmed K4b^2 ^by adding 1.7 kg to the subject's body mass. For each minute of the activity bout, the measured values were determined by the Cosmed K4b^2 ^and converted to kcal kg^-1 ^hr^-1 ^based on the conversion 1 MET = 1 kcal kg^-1 ^hr^-1^.

The 2006 C2RM [6] and refined C2RM [7] were used to predict EE using the ActiGraph GT1M accelerometer data. Both the C2RMs use an inactivity threshold, below which the subject is credited with 1.0 kcal kg^-1 ^hr^-1^. Both methods then examine the variability in counts and determine whether to use a continuous walk/run equation or intermittent lifestyle equation to predict EE (Table 1). A more detailed explanation of the refined C2RM is presented elsewhere [7]. In this study, we chose to express the EE values as one-minute averages, since nearly all researchers express their data in this manner [8-10].

**Table 1:** Description of the differences between the 2006 Crouter 2-Regression Model (2006 C2RM) and Refined Crouter 2-Regression Model (Refined C2RM)

Model	Inactivity Threshold	Determination of Variability	Determination of CV**	If CV ≤10%	If CV > 10%
2006 C2RM	50 counts min^-1^	six consecutive 10-s epochs	CV among 6 consecutive 10-s epochs	Walk/run equation	Intermittent lifestyle equation

Refined C2RM	8 counts per 10 s	Examines all combinations of surrounding five 10-s epochs*	Lowest CV out of all possible combinations	Walk/run equation	Intermittent lifestyle equation

*Examination includes 10-s epoch of interest and five before, 10-s epoch of interest and four epochs before and one after, etc.

**CV: coefficient of variation

### Statistics

Data analyses were performed using SPSS 16.0 for Windows (SPSS Inc., Chicago, IL). For all analyses, an alpha of 0.05 was used to denote statistical significance. The mean (± SD) values of predicted and measured kcal kg^-1 ^hr^-1 ^for all 10 participants in each condition (0-s, 20-s, 40-s) were computed. For each walking condition, an average kcal kg^-1 ^hr^-1 ^value was calculated for the Cosmed K4b^2 ^and the ActiGraph GT1M (2006 C2RM and refined C2RM). Repeated measures ANOVAs were used to statistically compare the average measured and predicted kcal kg^-1 ^hr^-1 ^values, for the total walking bout, for each condition. When appropriate, pairwise comparisons with Bonferroni adjustments were performed to locate significant differences.

To examine the validity of the 2006 and refined C2RMs during the transitions from rest to walking and back to rest, error scores (measured minus predicted kcal kg^-1 ^hr^-1^) were calculated for each minute, for each participant. A mixed model implemented as a two-way repeated-measures ANOVA was then used to analyze the data for each prediction equation, with condition being the among-participant factor and time (i.e., minute of the activity bout) being the within-participant factor. The interaction between condition and time was tested, and, in the cases of significant interaction, one sample t-tests were used to determine if the mean error score was significantly different from zero at each time point.

Bland-Altman plots [11] were created for each condition to graphically show the individual error between measured and predicted kcal kg^-1 ^hr^-1 ^values during the walking bouts, including the transitional minutes. This allowed for the mean error score and the 95% prediction interval (95% PI) to be shown. Prediction equations that are accurate will display a tighter prediction interval around zero. Data points below zero signify an overestimation, while data points above zero signify an underestimation.

## Results

Thirty (14 male and 16 female) participants completed the study (mean (± SD) age 28 ± 7.7 yrs; body mass index (BMI) 24.6 ± 3.6 kg m^-2^; 69% Caucasian, 17% African American, 7% Hispanic, 7% Other). Due to the differences in start times for each activity, different terminology was necessary. For the 0-s condition, the first minute of activity was referred to as "minute 1" with the remaining minutes labeled sequentially. For the 20-s and 40-s conditions, the first minute of the walking bout (i.e., transition from rest to walking) and the last minute of the bout (i.e., transition from walking to rest) were referred to as the first and last transitional minutes, respectively. The participants in these two conditions started and stopped walking 20 s and 40 s after the beginning of a minute on the ActiGraph clock, so the transitional minutes included time spent in both rest and activity. The minutes between the transitions consisted only of walking, and were labeled minutes 2-7. For example, a participant in the 20-s condition would have data for the first transitional minute, 7 full minutes of walking, and the last transitional minute.

**Figure 1:**
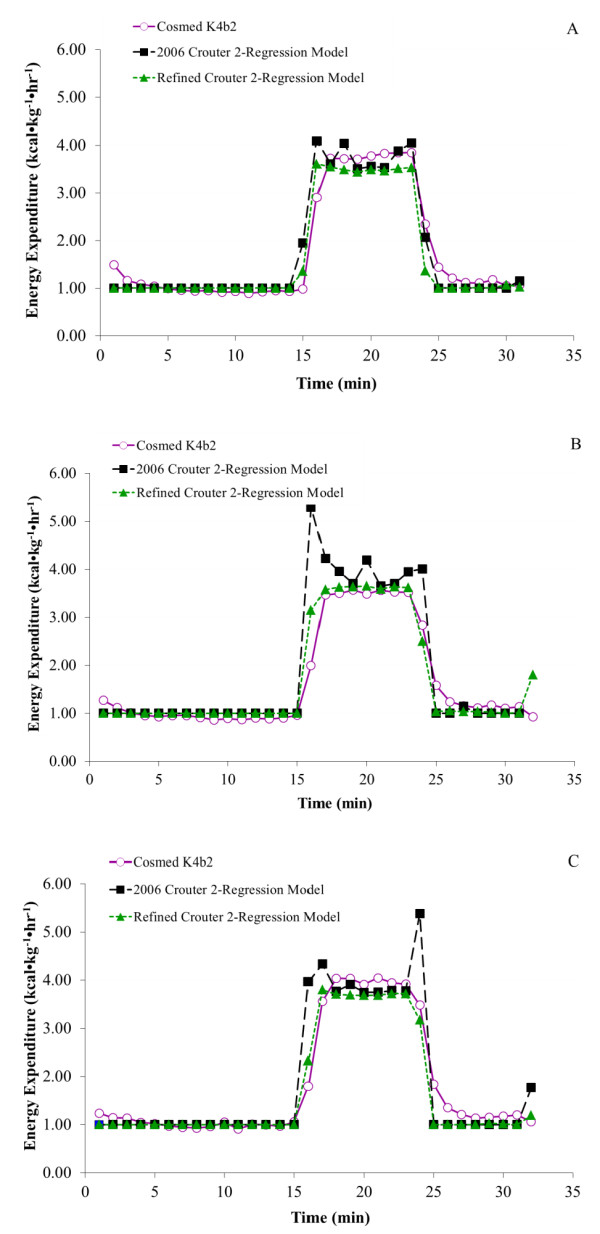
**Mean EE for each minute during a self-selected walking pace**. Mean measured (Cosmed K4b^2^) and predicted (2006 Crouter 2-Regression model (C2RM) and refined C2RM) energy expenditure for each minute of the: A) 0-s condition; B) 20-s condition; and C) 40-s condition during walking at a self-selected pace.

Figure 1 shows the measured and predicted EE values for each condition according to two different prediction equations. There was a significant interaction between time and condition for the walking bouts (P < 0.001), thus each condition was analyzed separately. Table 2 displays the mean (SD) energy cost during walking by condition. Except for the 0-s condition, the 2006 C2RM significantly over-predicted the mean energy cost (P = 0.02), while the refined C2RM was not significantly different from the mean measured energy cost (P > 0.05; Table 2). During the first minute of the 0-s bout, however, the 2006 and the refined C2RM significantly over-predicted measured EE by 1.2 ± 1.0 kcal kg^-1 ^hr^-1^and 0.7 ± 0.5 kcal kg^-1 ^hr^-1^, respectively (P < 0.01). These overestimates of the measured EE during the first minute of the 0-s bout were not significantly different from each other (p = 0.4). In addition, the 2006 C2RM and refined C2RM significantly over-predicted the measured EE in the first transitional minute in the 20-s condition by 3.3 ± 0.6 kcal kg^-1 ^hr^-1 ^and 1.1 kcal kg^-1 ^hr^-1^, respectively (P < 0.001). These over-predictions in the first transitional minute of the 20-s bout were significantly different from each other (P < 0.001). The 2006 C2RM also over-predicted the measured EE in last transitional minute of the 40-s condition (P < 0.001).

**Table 2:** Energy expenditure for the Cosmed K4b^2^, 2006 Crouter 2-Regression Model (2006 C2RM), and Refined Crouter 2-Regression Model (Refined C2RM) for each walking condition

Condition	Cosmed K4b^2^	2006 C2RM	Refined C2RM
All Conditions 0-s	3.6 (0.7) 3.7 (0.9)	4.0 (0.5)* 3.8 (0.9)	3.5 (0.5) 3.5 (0.5)
20-s	3.3 (0.7)	4.1 (0.9)*	3.4 (0.5)
40-s	3.6 (1.0)	4.0 (0.8)*	3.5 (0.6)

*Significantly different from measured kcal kg^-1 ^hr^-1 ^and refined C2RM (P < 0.02).

Energy expenditure expressed as kcal kg^-1 ^hr^-1 ^(Mean (SD)).

**Figure 2:**
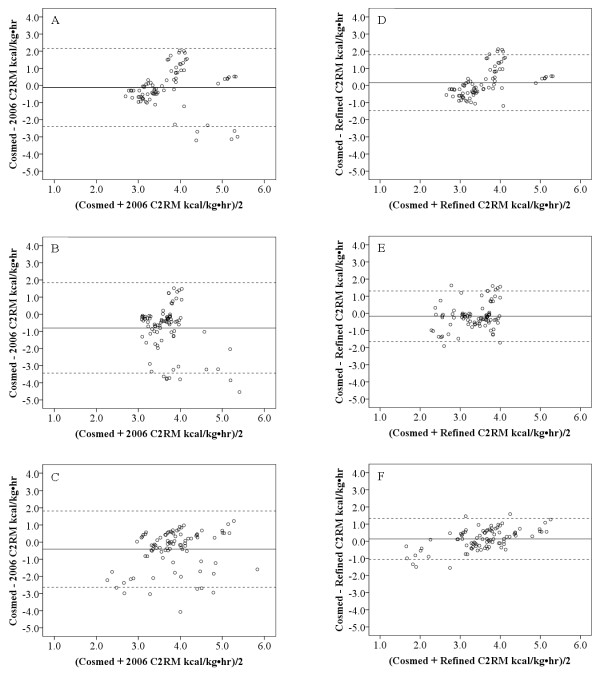
**Bland-Altman plots depicting error scores for the 2006 C2RM and the refined C2RM**. Bland-Altman plots depicting error scores (actual minus prediction) for each minute of walking, including transitional minutes for the 2006 Crouter 2-Regression Model (C2RM): A) 0-s condition, B) 20-s condition, C) 40-s condition, and the refined C2RM: D) 0-s condition, E) 20-s condition, and F) 40-s condition. The solid line represents the mean bias and the dashed lines represent the 95% prediction interval. Data points below zero signify an overestimation, while data points above zero signify an underestimation.

For each walking condition, the Bland-Altman plots (Figure 2) showed greater individual error for the 2006 C2RM compared to the refined C2RM. Specifically, for the 0-sec condition, the 2006 C2RM had a mean bias of -0.14 kcal kg^-1 ^hr^-1 ^(95% PI: -2.4, 2.2 kcal kg^-1 ^hr^-1^) and the refined C2RM had a mean bias of 0.16 kcal kg^-1 ^hr^-1 ^(95% PI: -1.5, 1.8 kcal kg^-1 ^hr^-1^). For the 20-s and 40-s conditions the 2006 and refined C2RM had similar mean bias and 95% PIs as what was seen for the 0-s condition.

## Discussion

There were two main findings of this study. First, the refined C2RM was a significant improvement over the 2006 C2RM and it closely reflected the measured kcal kg^-1 ^hr^-1 ^values, averaged over the entire bout, for all three conditions as hypothesized. Second, it was found that the refined C2RM predicted EE significantly better than the 2006 C2RM during transitional minutes from walking to resting.

Examining the first minute of the 0-s condition, there was a significant difference between the 2006 and refined C2RMs and measured EE. These differences resulted from the lag in oxygen uptake at the beginning of a bout (i.e., the "oxygen deficit"), as shown in Figure 1[12]. The predicted values of the first minute were similar to the measured steady state values, showing that both models accurately predict steady state EE values when the walking bouts start in synchronization with the ActiGraph clock. In contrast, during the first and last transitional minutes of the 20-s and 40-s conditions, the 2006 C2RM significantly over-predicted the measured kcal kg^-1 ^hr^-1^. The main reason is that the transitional minutes included some "0" counts in the beginning and end of the walking bouts, which increased the coefficient of variation (CV) within those minutes, causing the models to use the intermittent lifestyle equation. This led to an over-prediction of EE, which is especially evident in the first transitional minute of the 20-s bout and the last transitional minute of the 40-s bout.

The refined C2RM predicted EE more accurately than the original 2006 C2RM because it eliminated the over-predictions at the beginning and end of a walking bout without affecting the steady state values. Short (< 1 min), intermittent, walking bouts interspersed with inactivity would be classified as lifestyle activity, which was intentional. We chose not to identify bouts lasting less than 60 seconds as "walking" because brief periods of walking are often performed within the context of intermittent lifestyle activities (e.g., lawn-mowing, golf, and garden work). Thus, there is a risk that these lifestyle activities would be misclassified as walking, using that approach.

Based on the Bland-Altman plots, the refined C2RM provided a closer mean estimate than the 2006 C2RM. The one exception was for the 0-s condition where the 2006 and refined C2RM had similar mean errors; we expected this since the walking bout started exactly on the minute of the ActiGraph clock. For the 20-s and 40-s conditions the mean error was closer to zero for the refined C2RM due to it not being influenced by the transitions between walking and rest. In addition, for all walking conditions, the refined C2RM had tighter 95%PIs around the mean, indicating that it provided a more accurate individual prediction during structured walking bouts. An improvement in the individual prediction is an important step forward to obtaining more accurate predictions of free-living physical activity.

Recently Rothney and colleagues found that the 2006 C2RM significantly over-predicted EE measured by doubly-labeled water (DLW) and whole-room calorimetry by 6.0% and 10.2%, respectively [13]. Although we cannot be certain of what is causing this overestimation (since DLW does not capture minute-by-minute EE), the Rothney study did show the 2006 C2RM to overestimate moderate PA by 36.9 min compared to the whole-room calorimeter [13]. We hypothesize that the overestimations by the 2006 C2RM were likely due to the presence of multiple transitional minutes during their testing which would result in the misclassification of walking as intermittent, lifestyle activity, thus causing EE and time spent in moderate activity to be overestimated. Further, in a separate study we have shown that the refined C2RM significantly improved estimated EE and time spent in moderate activity during 6 hours of free-living measurement, compared to indirect calorimetry [14]. At this time it is difficult to quantify how much the refined C2RM improves upon the 2006 C2RM. During continuous walking bouts of long duration we would expect little improvement in the prediction of EE, since it is only the transitional minutes at the start and end of the bout that are being misclassified. However, individuals generally perform walking bouts of short duration which are mixed with intermittent lifestyle activities [15]. It was these shorter bouts of walking that were being misclassified resulting in an overestimation of EE and moderate activity. Future research should investigate the implications of these shorter bouts to be able to quantify the improvement of the refined C2RM compared to the 2006 C2RM in free-living environments.

A major strength of the current study is that we examined the time course of changes in measured and predicted kcal kg^-1 ^hr^-1^, rather than simply examining mean EE across the entire activity bout. This allowed us to examine the walk-to-rest transitions in detail. In addition, we were able to examine walking at a self-selected pace, which makes the results applicable to real-life settings.

The main limitation of this study is that we only validated the refined C2RM for walking bouts of 8 minutes duration, although we believe that continuous walking bouts as short as 1 minute can be identified using this technique. Future research is needed to determine the accuracy of the new, refined approach over longer time periods in free-living individuals, using doubly labeled water. Another limitation is that the 2006 C2RM was developed using the ActiGraph 7164, while the current study used the ActiGraph GT1M. Although different models were used, we do not feel it substantially influenced the results as it has been shown that the 7164 and GT1M give comparable count values during treadmill walking and running [16].

In conclusion, the 2006 C2RM significantly over-predicted EE during walking when the walking bout did not start in synchronization with the ActiGraph clock. The over-prediction of EE in the first and last minutes of the walking bouts for the 20-s and 40-s condition was due to the high CV during the transitional minutes. However, we observed that a refined C2RM [7] eliminated the over-predictions of EE that are likely to occur at the beginning and end of continuous walking bouts, and should therefore be used in future studies.

## List of abbreviations

EE: energy expenditure; C2RM: Crouter 2-Regression Model; MET: metabolic equivalent; BMI: body mass index; VO_2_: oxygen consumption; CV: coefficient of variation

## Competing interests

The authors declare that they have no competing interests.

## Authors' contributions

EK acquired data, assisted with analysis and interpretation, and was primarily involved in drafting and revising the current manuscript. SC made substantial contributions to the concept and design of the project, data analysis and interpretation, and has been involved with drafting and revising the manuscript. JH made a substantial contribution to the concept and design of the project, as well as, revisions to the current manuscript. EF made a substantial contribution to the concept and design of the project, as well as, revisions to the current manuscript. DB made substantial contributions to the concept and design of the project, data analysis and interpretation, and drafting and revising the current manuscript. All authors have given final approval of the version to be published.
